# AutoPepVax, a Novel Machine-Learning-Based Program for Vaccine Design: Application to a Pan-Cancer Vaccine Targeting EGFR Missense Mutations

**DOI:** 10.3390/ph17040419

**Published:** 2024-03-26

**Authors:** Enrico Bautista, Young Hyun Jung, Manuela Jaramillo, Harrish Ganesh, Aryaan Varma, Kush Savsani, Sivanesan Dakshanamurthy

**Affiliations:** 1 Georgetown University School of Medicine, Washington, DC 20007, USA; 2 Purdue University, West Lafayette, IN 47907, USA; 3 University of South Florida, Tampa, FL 33620, USA; 4 Virginia Commonwealth University, Richmond, VA 22043, USA; 5 The George Washington University, Washington, DC 20052, USA; 6 Lombardi Comprehensive Cancer Center, Georgetown University Medical Center, Washington, DC 20007, USA

**Keywords:** machine learning for peptide vaccine design, new vaccine design method, pan cancer vaccine, EGFR vaccine design, epitopes, MHC I and II, T-Cell receptors

## Abstract

The current epitope selection methods for peptide vaccines often rely on epitope binding affinity predictions, prompting the need for the development of more sophisticated in silico methods to determine immunologically relevant epitopes. Here, we developed AutoPepVax to expedite and improve the in silico epitope selection for peptide vaccine design. AutoPepVax is a novel program that automatically identifies non-toxic and non-allergenic epitopes capable of inducing tumor-infiltrating lymphocytes by considering various epitope characteristics. AutoPepVax employs random forest classification and linear regression machine-learning-based models, which are trained with datasets derived from tumor samples. AutoPepVax, along with documentation on how to run the program, is freely available on GitHub. We used AutoPepVax to design a pan-cancer peptide vaccine targeting epidermal growth factor receptor (EGFR) missense mutations commonly found in lung adenocarcinoma (LUAD), colorectal adenocarcinoma (CRAD), glioblastoma multiforme (GBM), and head and neck squamous cell carcinoma (HNSCC). These mutations have been previously targeted in clinical trials for EGFR-specific peptide vaccines in GBM and LUAD, and they show promise but lack demonstrated clinical efficacy. Using AutoPepVax, our analysis of 96 EGFR mutations identified 368 potential MHC-I-restricted epitope–HLA pairs from 49,113 candidates and 430 potential MHC-II-restricted pairs from 168,669 candidates. Notably, 19 mutations presented viable epitopes for MHC I and II restrictions. To evaluate the potential impact of a pan-cancer vaccine composed of these epitopes, we used our program, PCOptim, to curate a minimal list of epitopes with optimal population coverage. The world population coverage of our list ranged from 81.8% to 98.5% for MHC Class II and Class I epitopes, respectively. From our list of epitopes, we constructed 3D epitope–MHC models for six MHC-I-restricted and four MHC-II-restricted epitopes, demonstrating their epitope binding potential and interaction with T-cell receptors. AutoPepVax’s comprehensive approach to in silico epitope selection addresses vaccine safety, efficacy, and broad applicability. Future studies aim to validate the AutoPepVax-designed vaccines with murine tumor models that harbor the studied mutations.

## 1. Introduction

Identifying immunodominant epitopes to elicit clinically significant responses is crucial in developing a successful peptide vaccine. Nevertheless, the selection of epitopes in previous in silico studies has typically only relied on predicting the binding affinity of the epitopes to a patient’s HLA molecules [1,2]. Therefore, there is a need to improve the epitope selection process for peptide vaccine design. Our previous studies have enhanced the epitope selection methods by applying exclusion criteria based on several epitope characteristics [3,4,5]. These methods required some manual data collection across several online tools, and they did not utilize experimental data to determine the cutoffs for the exclusion criteria [3,4,5]. Another study by Gartner et al. used experimental data to train a machine-learning-based model that ranks MHC-I-restricted epitopes based on computational epitope characteristics and transcriptomic data [6,7]. The model developed by Gartner et al. is based upon tumor neoantigen data. However, this model can only be used for the design of personalized peptide vaccines, as it requires RNA sequence analysis data [7]. Additionally, Gartner et al. did not consider MHC-II-restricted epitopes or safety precautions such as toxicity and allergenicity in their model [7]. While these studies have improved upon epitope selection efforts, there are no widely accepted methods of epitope selection that automatically rank epitopes based on the epitope sequence alone. Such a method would allow preclinical and clinical peptide vaccine trials to efficiently prioritize the testing of epitopes without requiring tumor samples or RNA sequence analysis data. Here, we applied our AutoPepVax program to select neoantigens derived from common EGFR missense mutations across four cancers. Then, we assessed the efficacy of a pan-cancer vaccine targeting the EGFR neoantigens identified by AutoPepVax.

A pan-cancer vaccine is a vaccine that aims to target several cancers by introducing the immune system to epitopes derived from common tumor antigens across several cancers. Many clinical trials assessing the efficacy of peptide vaccines for cancer therapy are underway [1,8,9]. However, no clinical trials are investigating pan-cancer peptide vaccines. Peptide vaccines targeting tumor-specific antigens (TSAs) often include mutated epitopes from a common mutant protein. Mutant epidermal growth factor receptor (EGFR) has been a target for peptide vaccines [1,8,9,10,11,12]. EGFR mutations drive tumorigenesis through the MAPK and PI3K signaling pathways for many cancers, including lung adenocarcinoma (LUAD), glioblastoma multiforme (GBM), colorectal adenocarcinoma (CRAD), and head and neck squamous cell carcinoma (HNSCC) [3,4,5]. A phase III study (NCT01480479) of a vaccine containing deletion mutation EGFRvIII-specific peptides concluded that the vaccine did not improve survival for newly diagnosed GBM patients [9,11]. However, patients receiving the same vaccine administered concurrently with granulocyte–macrophage colony-stimulating factor in a phase II study (NCT01498328) showed robust anti-EGFRvIII antibody titers and potential therapeutic benefits [8,10]. A phase I study (NCT04397962) of personalized peptide vaccines targeting mutant EGFR in non-small-cell lung cancer (NSCLC) demonstrated that the L858R and T790M mutations were immunogenic in four patients [1,12]. Vaccinations in murine models of EGFR mutant NSCLC also demonstrate immune responses against tumors [13,14]. Evidence of peptide vaccines eliciting anti-tumor responses in GBM and NSCLC suggests the potential for the development of robust vaccines for other EGFR-mutant cancers, including LUAD, GBM, CRAD, and HNSCC.

EGFR mutations are present in 38.0% of patients with LUAD. LUAD accounts for 40% of all lung cancer diagnoses. Pembrolizumab and nivolumab can prolong the 5-year survival rates of patients with LUAD to nearly 25% [15]. Standard interventions for GBM, such as surgery, radiation, and systemic therapy, can only extend the patient’s overall survival by several years [16]. GBM accounts for nearly 80% of all brain cancer cases and has a median survival period of roughly 14–15 months [17]. Among patients with GBM, the EGFRvIII deletion mutation is the most prevalent [18]. Head and neck squamous cell carcinoma (HNSCC) has an EGFR mutation frequency of 80–90% [19]; hence, recent immunotherapeutic treatments have mainly targeted EGFR. Pembrolizumab and nivolumab improve the prognosis for patients with HNSCC [19]. CRAD is the second leading cause of cancer death worldwide, with an overall 5-year survival rate of just over 60% [20]. Current therapies include surgery, chemotherapy, radiation, and EGFR inhibitors when warranted [21]. Peptide vaccines for CRAD are hindered by adverse immunosuppression and intolerance [22]. EGFR is overexpressed in around 70% of cases, and the mutations may be oncogenic [21]. EGFR mutations are prevalent in each of these cancers; therefore, patients with these cancers may benefit from a pan-cancer vaccine that targets common EGFR mutations. However, determining the epitope composition for a vaccine remains a challenge. In this study, we developed the Python-based AutoPepVax version 1.0 software program to select and rank candidate epitopes for inclusion in a peptide vaccine. We applied AutoPepVax to the design of the first pan-cancer vaccine design to target EGFR, and our results indicate that the EGFR missense mutations for each cancer contain immunogenic peptides. AutoPepVax could be a starting point for preclinical vaccine research by automating the prioritization of candidate neoantigens.

## 2. Results

### 2.1. Workflow of Results

**Figure 1:**
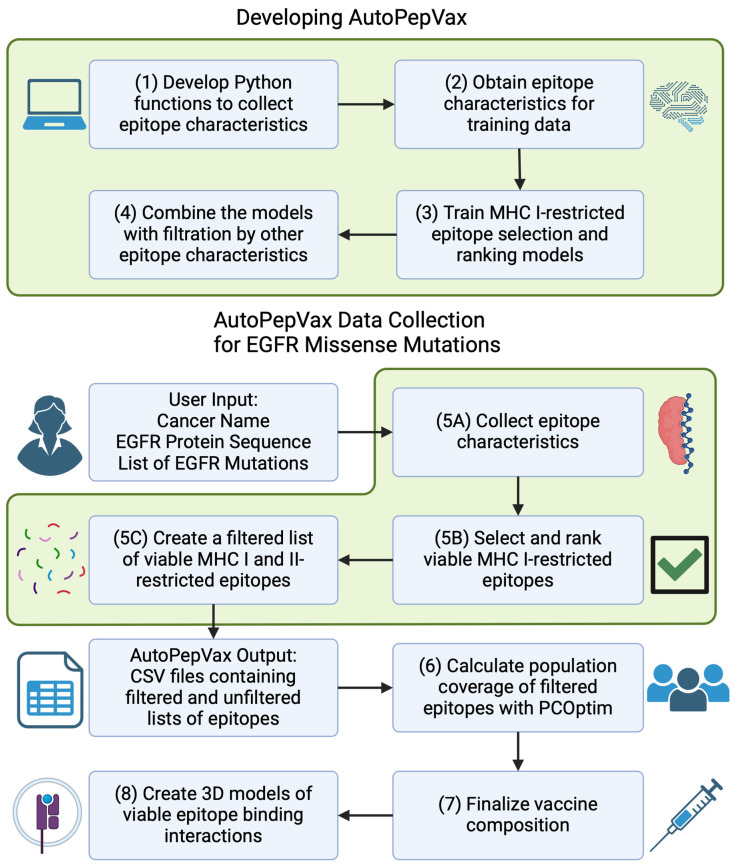
Workflow of the AutoPepVax design and application of AutoPepVax to pan-cancer vaccine design targeting EGFR. (1) Develop Python functions to automatically collect characteristics of mutated epitopes, such as binding affinity to HLA molecules and antigenicity. The characteristics are calculated by various in silico tools. The outputs of the in silico tools are collected by web scraping or using an API. (2) Obtain a dataset of MHC-I-restricted epitopes from tumor samples known to induce TILs or be non-antigens and determine their characteristics. (3) With the dataset, train a random forest model to classify the epitopes as positive (lymphocyte-inducing) or negative (non-antigen) epitopes. In addition, train a linear regression model to score the epitopes so that they may be ranked within their respective groups. (4) Combine the classification and regression model with the exclusion criteria from our prior studies into one program. (5A) Find EGFR missense mutations commonly implicated in several cancers. Use AutoPepVax’s data collection capabilities to determine the values of various epitope characteristics for the mutated epitopes. (5B) AutoPepVax classifies and ranks MHC-I-restricted epitopes with our models. (5C) AutoPepVax filters the lists of viable epitopes via exclusion criteria. The filtered and unfiltered lists are output by AutoPepVax as CSV files to a folder specified by the user. (6) Use PCOptim to determine the population coverage of a vaccine with a minimal epitope formulation based on the AutoPepVax output. (7) Using the epitope list generated by PCOptim and finalize the pan-cancer vaccine composition, which contains mutated MHC-I- and II-restricted epitopes. (8) Create 3D models of some viable epitopes bound to TCRs and HLA molecules.

AutoPepVax employs machine-learning-based models for EHLA-I pair selection and ranking, as described in Figure 1. We trained and assessed the RF classification and linear regression models incorporated in AutoPepVax with five-fold cross-validation. Other classification and regression models were similarly validated to compare their performance to that of the RF and linear regression models. After validating the models, AutoPepVax was applied to a list of prevalent EGFR missense mutations in four different cancers. AutoPepVax produced filtered lists of positive EHLA-I and EHLA-II pairs from the EGFR mutations. From these lists, PCOptim-CD [3] was used to determine the population coverage of the positive EGFR EHLA pairs. Finally, we constructed 3D models of select EGFR EHLA complexes and EHLA–TCR complexes.

### 2.2. Validating AutoPepVax’s MHC-I-Restricted Epitope Assessment Model

**Figure 2:**
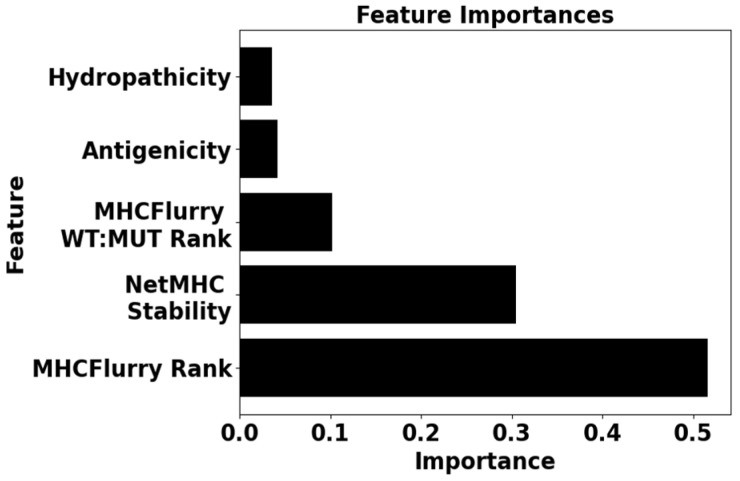
Here, the feature importances of the random forest model, which classifies EHLA-I pairs, are displayed in a bar graph. The levels of importance correspond to the relative contributions of each feature to the classification of EHLA-I pairs.

The dataset used to train the random forest classification and linear regression models used in AutoPepVax included 763 EHLA-I pairs. Of these pairs, 109 were labeled as positive or able to induce tumor-specific CD8+ T cells. The other 654 pairs were negative or unable to induce tumor-specific CD8+ T cells. RF classification models with different combinations of input features were assessed via five-fold validation. The average accuracy scores, PPV, and TPR for each validation can be seen in Supplementary Table S1. The RF classification model with the input features of antigenicity, hydropathicity, MHCflurry rank, MHCflurry wild-type rank to mutant rank ratio, and NetMHCstabpan stability prediction offered the most accurate model (accuracy = 0.96). AutoPepVax used this model to classify the EHLA-I pairs as positive or negative. The relative importance of each feature in this random forest model is shown in Figure 2. We used the RF classification model to classify the epitopes used in peptide vaccine clinical trials. We classified the EHLA-I pairs as positive or negative for 103 epitopes used in clinical trials. The RF classification model identified 62.1% of the epitopes as having at least one positive EHLA-I pair.

To compare different classification models, we performed cross-validations for the SVM, GNB, and MLP classification models with different combinations of input features. The MLP classification model with the input features of antigenicity, hydropathicity, MHCflurry rank, MHCflurry wild-type rank to mutant rank ratio, and NetMHCstabpan stability prediction offered the second most accurate model (accuracy = 0.967). This model had the best TPR, with 0.928 of the identified positive EHLA-I pairs being true positives. The best RF classification model had a TPR of 0.898. The least accurate model was the SVM classification with the input features of immunogenicity, hydropathicity, NetMHC rank, MHCflurry wild-type rank to mutant rank ratio, and NetMHCstabpan stability (accuracy = 0.950). Two GNB classification models had the lowest TPRs (TPR = 0.752). The average accuracy scores, PPV, and TPR for these cross-validations can be seen in Supplementary Table S1.

To further sort the positive pairs selected by the RF classification model, we trained and tested a linear regression model to score the EHLA-I pairs via five-fold validation. Again, different combinations of input features were tested to determine which input features provided the best model. The average AUC of ROC for the folds of each validation can be seen in Supplementary Table S2. Each linear regression model had an AUC greater than 0.980 regardless of the input features used. Of the tested linear regression models, the model with the input features of MHCflurry rank, MHCflurry wild-type rank to mutant rank ratio, and NetMHCstabpan stability had the highest AUC of ROC (AUC = 0.989). This linear regression model was used to rank the positive EHLA-I pairs for each cancer.

We also validated the RF, GB, and logistic regression models. The logistic regression model with the input features of MHCflurry rank, MHCflurry wild-type rank to mutant rank ratio, and NetMHCstabpan stability had the highest AUC of ROC (AUC = 0.992) of all models. When we attempted to rank the test epitopes by score using this linear regression model, most epitopes scored exactly 1 or 0. These epitopes could not be ranked because their scores were too close to compare. The GB regression models had the lowest AUCs, ranging from 0.953 to 0.976. All other models had AUCs higher than 0.976. The average AUC of ROC for the folds of these cross-validations can be seen in Supplementary Table S2.

### 2.3. AutoPepVax Operation

**Figure 3:**
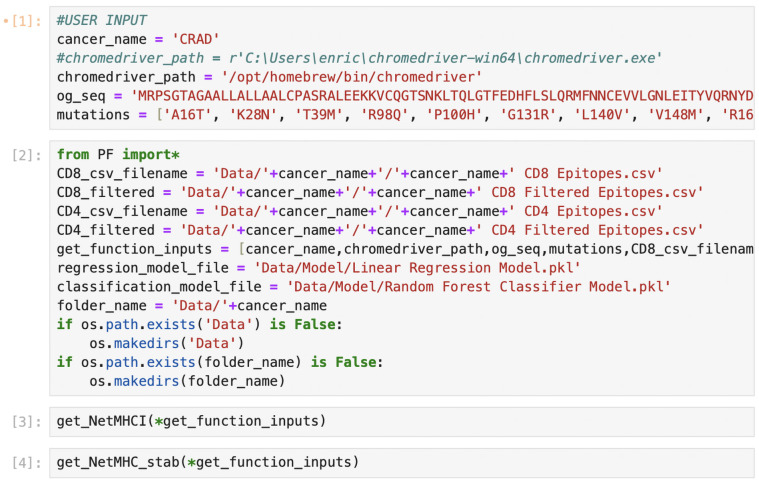
An excerpt of the Jupyter Notebook through which AutoPepVax’s programs can be run to collect data for CRAD missense mutations. In the first cell, the user alters the variables to specify the name of the cancer in which the mutations occur, the canonical sequence of the protein, and the missense mutations to be analyzed. All cells beneath do not need to be adjusted. The user may simply run all cells to generate the output files.

AutoPepVax is run on a Jupyter Notebook. It requires several dependencies. A full description of how to install and run AutoPepVax is freely available at Https://Github.Com/Enricobautista/Autopepvax (accessed on 21 March 2024). Once the user has opened the Jupyter Notebook that runs AutoPepVax and installed the proper dependencies, the user must specify a cancer name, an unmutated protein sequence, and a list of missense mutations for AutoPepVax to analyze. The user can do this by altering the variables in the top cell of the Jupyter Notebook, as shown in Figure 3. After the inputs are defined, the user can run all of the cells, and AutoPepVax will analyze all possible mutant epitopes as described in the Methods. Each cell will be run sequentially. The cell currently being executed is indicated by a star in brackets on the left side of the cell, as follows: [*]. Completed cells will be numbered within brackets on their left, as shown in Figure 3. Upon the execution of all cells, AutoPepVax populates a folder with the cancer name that contains filtered and unfiltered EHLA pair lists. The output files are described in Table 1. The user can access this folder via the same folder that AutoPepVax was run from.

### 2.4. EGFR-Mutated Epitopes Identified by AutoPepVax

Across GBM, CAC, LUAD, and HNSCC, 96 missense mutations were analyzed. Among EHLA-I pairs, 49,113 were analyzed, yielding 368 positive pairs after filtration. Among EHLA-II pairs, 168,669 were analyzed, yielding 430 positive pairs after filtration. The mutations studied for each cancer and the total number of EHLA pairs before and after filtering are shown in Supplementary Table S3. Supplementary Tables S4–S19 include the complete lists of EHLA pairs and their characteristics. Table 2 lists the mutations that had positive EHLA-I and EHLA-II pairs. Of the 96 missense mutations assessed, only 19 had positive EHLA-I and EHLA-II pairs. The G598V mutation is the only one of these 19 mutations to be prevalent in multiple cancers.

### 2.5. Pan-Cancer Vaccine Population Coverage

For all positive EHLA pairs of each cancer, the world and regional population coverage was determined. The population coverage calculations of EHLA-I pairs and EHLA-II pairs for each region are shown in Supplementary Tables S20–S23. The optimized epitope lists used to find the population coverages are included in Supplementary Tables S24 and S25.

### 2.6. Population Coverage

The combined dataset of CRAD, LUAD, HNSCC, and GBM EHLA-I pairs had optimal world population coverage of 98.55%. EHLA-I pairs had average regional coverage of 89.03%. Europe had the highest regional coverage at 99.68%, and Central America had the lowest coverage at 7.76%. The world and regional coverages for EHLA-I are shown in Supplementary Tables S20 and S21. The average number of epitopes in the EHLA lists that were recognized by people around the world, or the average epitope/HLA combination hits, was calculated. The minimum epitope/HLA combination hits recognized by 90% of the world was also calculated. The filtered set of EHLA-I pairs have an average of 22.66 epitope/HLA combination hits recognized by the population. Of these, 5.32 epitope/HLA combination hits are recognized by a minimum of 90% of the population per region.

The filtered EHLA-II pairs resulted in the total world coverage of 81.81% with an overall 37.84 average hits. EHLA-II pairs had average regional coverage of 64.55%. North America had the highest regional coverage at 87.89% and South Africa had the lowest coverage at 32.10%. Due to the limitations of the IEDB database, not all EHLA-II pairs could be assessed for population coverage. A list of all of the EHLA-II pairs that were not accepted through IEDB and, therefore, were excluded from the population coverage results is listed in Supplementary Table S26. The world and regional coverages between the optimized and filtered sets were equal for the pan-cancer dataset. Therefore, no exclusion criteria from AutoPepVax hinder the population coverage. The world population coverage data for each list of epitopes can be seen in Table 3.

### 2.7. TCR Models and Binding

**Figure 4:**
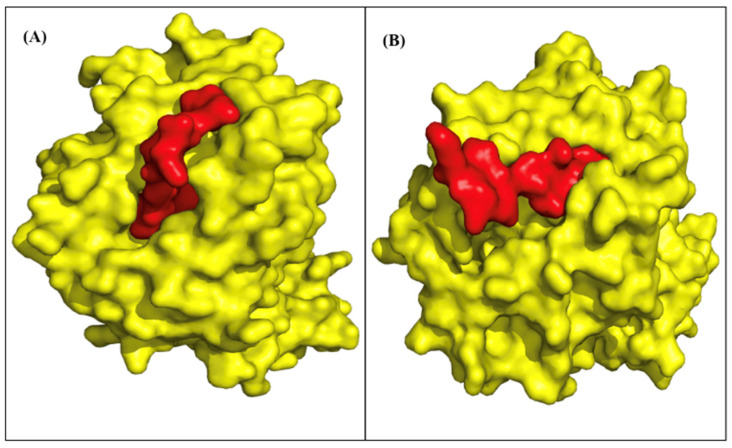
The yellow structures are HLA molecules, and the red structures are epitopes. (**A**) Glioblastoma multiforme epitope, VVMGENNTLV, binding to MHC Class I molecule HLA-A*02:06 (RCSB PDB: 3OXR). (**B**) Head and neck squamous cell carcinoma epitope, ILKETELKK, binding to MHC Class I molecule HLA-A*03:01 (RCSB PDB: 7L1C, A chain). These models appear to illustrate epitopes binding efficiently to the binding grooves of the MHC molecule.

**Figure 5:**
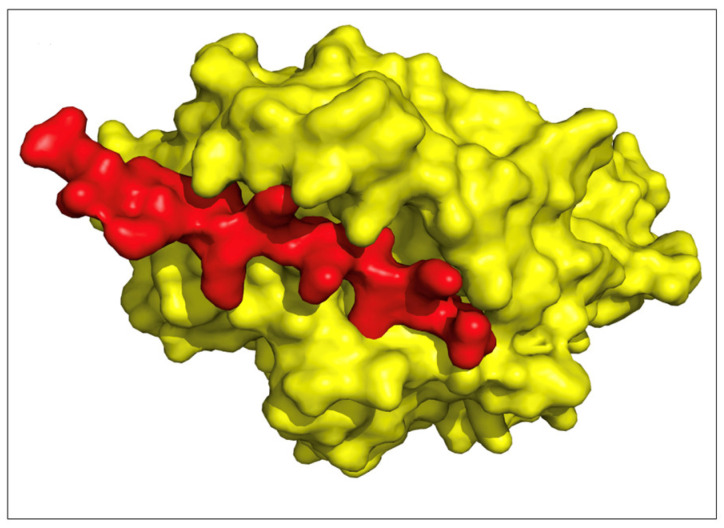
The yellow structure is the HLA molecule, and the red structure is the epitope. The figure shows 3D models for the EHLA pairs of a CD4 top epitope. Colorectal adenocarcinoma epitope, ILKKTEFKKIKVLGS, binding to MHC Class II molecule HLA-DRB1*04:01 (RCSB PDB: 5JLZ).

**Figure 6:**
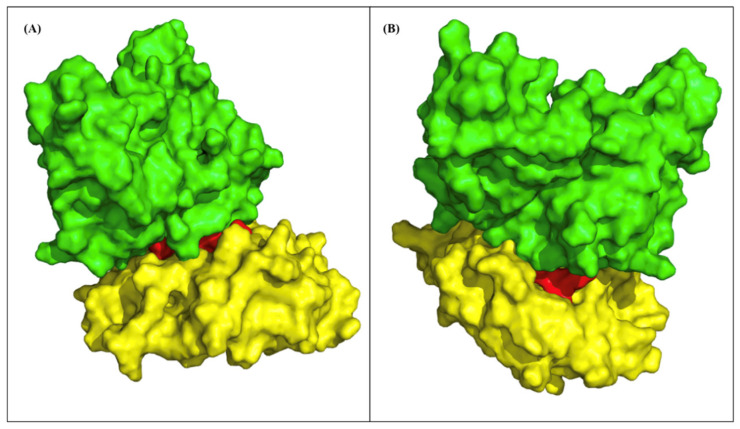
The yellow structures are HLA molecules, and the red structures are epitopes. The green structures are TCRs. (**A**) ILKETELKK-HLA-A*02:06 complex binding to the human 0606T1-2 TCR complex (RCSB PDB: 7RRG, C and D chain). (**B**) ILKETELKK-HLA-A*03:01 complex binding to the D30 TCR in the complex. The EHLA complexes appear to bind effectively to the TCR complex.

We modeled three top epitopes to two EHLA-I pairs and one EHLA-II pair using mDockPep and PyMol. The 3D models of the EHLA pairs are shown in Figure 4 and Figure 5. The bound epitopes are docked within the binding grooves of the HLA molecules. Then, the 3D models of the EHLA–TCR interactions were developed utilizing TCRModel, as shown in Figure 6. The 0606T1-2 TCR complex is sensitive to HLA-A*03 and was thus used to model the interactions between the TCR complex and HLA-A*03:01 bound to ILKETELKK. The TCRs are able to dock closely to the EHLA pairs such that the residues of the TCRs interact with the epitope. Supplementary Figures S1–S3 include superimposed images of our EHLA pairs with sample HLA molecules from the RCSB Protein Data Bank. The EHLA complex aligns closely with the original protein in the superimposed images. We did not find any significant conformation deformation of the HLA with the peptide epitope’s bound structure. The 3D coordinates of all pMHC and pMHC–TCR models’ PDB files for Figure 4, Figure 5 and Figure 6 and Supplementary Figures S1–S3 are available for download in the Supplementary PDB Files.

## 3. Discussion

We developed AutoPepVax to automatically select immunogenic EHLA pairs that do not cause allergic or toxic effects. Our prior methods of epitope selection address the safety and efficacy of vaccine candidates via a series of exclusion criteria [3,4,5]. However, the lists that we generated with these methods did not rank EHLA pairs [3,4,5]. Therefore, relying on exclusion criteria alone does not provide a definitive method to prioritize epitope testing in pre-clinical and clinical trials. Gartner et al. addressed this issue by developing a model for the ranking of tumor neoantigens that is trained on experimental data derived from tumor samples [7]. This model requires RNA sequence analysis data and is geared toward the development of personalized cancer vaccines [7]. Gartner et al. also did not consider the viability of EHLA-II pairs, a primary limitation of their study [7]. Thus, unlike the model developed by Gartner et al., AutoPepVax can rank viable EHLA-I pairs or select viable EHLA-II pairs without the need for tumor-specific RNA sequence analysis data [7]. The levels of neoantigen expression may vary across tumors, so cancer-specific and pan-cancer vaccine studies cannot consider tumor-specific transcriptomic data. Additionally, the model developed by Gartner et al. does not include the safety precautions that our previous methods employed [3,4,5,7]. AutoPepVax applies the strengths of Gartner et al.’s epitope selection model by utilizing two machine-learning-based models, a linear regression model and an RF classification model, with subsequent filtration by exclusion criteria. Both models are validated with experimental data. By training the models on known positive EHLA-I pairs, our models have a concrete threshold to determine whether an EHLA-I pair will induce TILs and a basis for the ranking of EHLA-I pairs. With AutoPepVax, potential peptide-based cancer vaccines targeting many missense mutations can be studied expeditiously. AutoPepVax may be applied to pan-cancer, cancer-specific, and personalized peptide vaccines. For pan-cancer and cancer-specific peptide vaccines, the user must input any mutations of interest and AutoPepVax will rank the viable epitopes. The user input can be adjusted to study mutations and HLA alleles that are specific to a patient; studies aiming to develop personalized peptide vaccines may produce ranked EHLA-I lists for each participant.

We trained our models with five-fold cross-validation to reduce the possibility of overfitting our models to one subset of the data that we used. We found that the linear regression model was robust, with an AUC of 0.989. However, this linear regression model was not the highest-performing regression model. Of all validated models, the logistic regression model with the input features of the MHCflurry rank, MHCflurry wild-type rank to mutant rank ratio, and NetMHCstabpan stability had the highest AUC of ROC (AUC = 0.992). Despite its impressive AUC, this logistic regression model generates mostly binary scores of 0 or 1 for EHLA-I pairs. These binary scores were too similar to compare. Therefore, this model is unfit for the ranking of EHLA-I pairs. Of the classification models validated, AutoPepVax’s RF classification model was the most accurate (accuracy = 0.967). It is also important to consider the model sensitivity, as only a small portion of the analyzed EHLA pairs will be immunogenic. The RF classification model had a TPR of 0.898. Only the MLP classification model with the input features of antigenicity, hydropathicity, MHCflurry rank, MHCflurry wild-type rank to mutant rank ratio, and NetMHCstabpan stability prediction offered a better TPR (TPR = 0.928). With either model, less than 11% of the truly positive EHLA-I pairs will be falsely labeled as negative. Thus, AutoPepVax will not egregiously omit therapeutic epitopes. The RF classification model was tested on epitopes that were used in peptide vaccine clinical trials. Despite its accuracy during the five-fold validation, the RF classification model identified only 62.1% of the epitopes from clinical trials as having at least one positive EHLA-I pair. We did not investigate whether these epitopes successfully induced anti-tumor immune responses. It is possible that the TAAs used in clinical trials are not highly immunogenic.

According to the feature importance of our RF model, the binding rank, wild type to mutant rank ratio, binding stability, antigenicity, and hydropathicity were determined to be important in ranking and classifying EHLA-I pairs. These features have previously been used by us and others to assess epitope viability for peptide vaccines [3,4,7]. The general trends of the linear regression model scores indicate this as well. EHLA-I pairs with high scores tend to have a low binding rank and high stability. It is uncommon for a positive EHLA pair to have a binding rank above the fifth percentile. Despite the binding rank being an important parameter, the ratio of the wild type to mutant epitope rank carries much less importance. It may be that substituting a single amino acid due to a missense mutation does not usually alter the binding properties of an epitope significantly. Although hydropathicity and antigenicity contribute some value to the classification of EHLA-I pairs, they demonstrated the least importance of the five input features for the RF classification. These characteristics tend to vary the most among the positive EHLA-I pairs.

We applied AutoPepVax to the design of a pan-cancer peptide vaccine targeting EGFR missense mutations. AutoPepVax was used to determine the positive EHLA-I and EHLA-II pairs for prevalent EGFR mutations across various cancers. AutoPepVax’s inclusion of EHLA-II pairs accounts for the various effects that CD4+ T cells have on the antitumor immune response [23,24]. There were 19 mutations with both positive EHLA-I and EHLA-II pairs. The G598V mutation was represented in both the GBM and LUAD vaccines. Immunization with peptides specific to G598V mutations may elicit a particularly potent immune response against cancers harboring this mutation.

After selecting the positive EGFR EHLA-I and EHLA-II pairs, we used PCOptim-CD to determine their population coverage [3]. The optimized lists of EHLA-I pairs retained population coverage of 98.55%, and the list of EHLA-II pairs retained coverage of 81.81%. The EHLA-I pairs had average regional coverage of 89.03%. However, Central America had exceedingly low coverage at 7.76%. No other regional coverages were lower than 80%. Central America, in this coverage calculation, only includes Costa Rica and Guatemala [25]. In Central America, only 12.38% of the population carry HLA-A or HLA-B alleles [25]. Because the binding characteristics were calculated exclusively with HLA-A and HLA-B molecules, prevalent HLA alleles of the Guatemalan and Costa Rican populations were not included in our initial analysis of EHLA-I pairs [25]. With each EHLA pair dataset resulting in high population coverage, AutoPepVax provided a thorough filtering process for epitope selection. Additionally, there were no population strains imposed by AutoPepVax’s process of epitope selection. Although the filtered and optimized epitope lists had equal coverage, the filtered epitopes had a much higher average epitope hits per allele. Thus, AutoPepVax often identified several distinct EHLA pairs for each HLA allele. Having several epitopes identified by a single HLA molecule indicates that one mutation is particularly immunogenic for carriers of this HLA allele or several mutations can induce TILs through this HLA molecule. Both implications are beneficial for vaccine design. This confirms that AutoPepVax selected a robust and extensive list of EHLA pairs.

While our results are promising, displaying the binding properties that we calculated with 3D models is helpful. We created models of three EFGR EHLA complexes from the optimized lists. In all of the models, the epitope fits within the HLA molecule. Then, we superimposed the original structures of the HLA molecules onto the EHLA complexes. The original HLA molecule had no noticeable conformation deformation in the superimposed models. Thus, the epitopes in these pairs truly bind the HLA molecules that they are paired with. For the two EHLA-I complexes, we created EHLA–TCR complex models. The TCRs were able to dock in close proximity to the EHLA pair complexes. Additionally, none of the epitopes demonstrated clashing with the TCRs. These findings confirm the possibility of these epitopes being able to bind HLA molecules and be recognized by a TCR.

## 4. Limitations

AutoPepVax is capable of automatically generating a list of EHLA pairs that are derived from missense mutations. Missense mutations are relevant to pan-cancer, cancer-specific, and personalized peptide vaccine studies. However, plenty of malignancies are characterized by frameshift, insertion, or deletion mutations. Tumors harboring these classes of mutations may not be studied with the current version of AutoPepVax. Consequently, we were unable to study the EHLA pairs generated by the EGFR deletion mutation that is common in GBM [26]. The inability to analyze other mutation classes reduces AutoPepVax’s applications to the study of cancers carrying missense mutations.

AutoPepVax’s selection and ranking of EHLA-I pairs rely on RF classification and linear regression models. The data used to train these models included epitopes that were recognized by TILs and non-immunogenic antigens. Thus, the models predict an epitope’s ability to induce TILs. Although the induction of TILs is an important step in an anti-tumor immune response, it cannot be assumed that all induced TILs will lead to therapeutic benefits. Due to data availability limitations, we could not train our models with clinical significance as an endpoint. Thus, no conclusion can be made about the therapeutic effects of the EHLA-I pairs selected by AutoPepVax.

We used PCOptim-CD [3] to calculate the population coverage of the positive EGFR EHLA pairs identified by AutoPepVax. The HLA alleles that can be included in the population coverage calculations are dictated by the population data available on IEDB. Our calculations were limited by the MHC Class II population data, as only 57% of the EHLA-II pairs could be assessed. This led to an underestimate of the population coverage for EHLA-II pairs.

Despite the limited population data for EHLA-II pairs, the positive EHLA-I and EHLA-II pairs could achieve sufficient world population coverage. However, there is little overlap in the mutations specific to each cancer. Only the G598V mutation was identified as a mutation that produces positive EHLA-I and EHLA-II pairs for more than one cancer. For patients to benefit from a vaccine, inoculated peptides must address their specific tumor mutations and match their HLA phenotypes. Therefore, a pan-cancer vaccine would likely include many peptides that are irrelevant to the patient receiving it. Depending on the manufacturing practices for vaccines, it might be more sensible to produce a separate vaccine for each cancer or personalized vaccines as needed.

## 5. Future Directions

We will continue to develop AutoPepVax to improve its ease of use. AutoPepVax is currently executed within the JupyterLab environment, so those who are unfamiliar with Python may have difficulty operating AutoPepVax. We will create a graphical user interface (GUI) to allow for the more intuitive use of AutoPepVax. Our GUI will be simple to download and operate.

As mentioned in the Limitations, non-missense mutations may not be analyzed by AutoPepVax. Given that numerous cancers are defined by various classes of mutations, we will update AutoPepVax to accommodate the analysis of frameshift or deletion mutations for these cancers. Personalized vaccine studies may use transcriptomic data to inform epitope selection for patients. We will develop models that utilize transcriptomic data for the study of specific tumor samples in personalized vaccine trials.

With these updates, AutoPepVax will have more use cases in investigating personalized peptide vaccines and other cancers. In one such case, we plan to retrospectively investigate whether AutoPepVax can predict whether immunized epitopes improve patient clinical outcomes. We will collect a list of EHLA pairs from past clinical trials and determine whether AutoPepVax can scrutinize which of the EHLA pairs causes tumor regression or improved survival.

We also aim to substantiate AutoPepVax’s efficacy with preclinical trials. With 368 EHLA-I pairs and 430 EHLA-II pairs identified as positive, there are many permutations for possible EGFR-specific vaccines. We will administer our vaccine to murine models using high-scoring EGFR EHLA pairs from each cancer. Dosage–response testing and CD4+ and CD8+ T-cell responses will be used to characterize the potency and efficacy of our vaccine. We will also further investigate the characteristics of the epitopes used in clinical trials that were not identified by AutoPepVax’s RF classification model.

## 6. Conclusions

In this study, we developed AutoPepVax to expedite and improve the in silico epitope selection for peptide vaccine design. AutoPepVax takes the input of missense mutations for a given protein and identifies mutant epitopes that will safely induce TILs. AutoPepVax is the first automated neoantigen ranking method trained with tumor neoantigen data that can assist in the study of pan-cancer, cancer-specific, and personalized peptide vaccines. Preclinical trials can be expensive and arduous, so AutoPepVax offers a simple solution to narrow candidate epitopes for the design of pan-cancer, cancer-specific, and personalized peptide vaccines. AutoPepVax leverages two machine-learning-based models: a linear regression and an RF classification model. The linear regression model that we developed is used to rank EHLA-I pairs based on their ability to induce TILs. The linear regression model is adept at making ranking predictions by using the binding characteristics. The AUC of this model’s ROC was 0.989. The linear regression model scores for EHLA-I pairs determine which epitopes to prioritize for efficient testing in preclinical and clinical trials for peptide vaccines. By ranking EHLA-I, AutoPepVax allows experimenters to assess epitopes in vitro or in vivo in the most cost-effective manner. This is far more robust than relying on the binding rank calculated by a single online tool. The RF classification model that we developed separates EHLA-I pairs into positive and negative groups with accuracy of 0.967. This model is sensitive to epitope selection, as it had a TPR of 0.898. The RF classification model will likely identify epitopes that induce TILs. We have combined the capabilities of these models with exclusion criteria to enhance the safety precautions of AutoPepVax’s epitope selection.

We used AutoPepVax to develop an EGFR-specific pan-cancer vaccine design for GBM, LUAD, HNSCC, and CRAD. Our analysis of 96 missense mutations with AutoPepVax yielded 368 positive EHLA-I pairs and 385 positive EHLA-II pairs. The pan-cancer EHLA pair lists had maximum world population coverage of 98.55% for EHLA-I pairs and 81.81% for EHLA-II pairs. Because the HLA alleles for 43% of the EHLA-II pairs were not included in IEDB’s population coverage tool, the population coverage for EHLA-II pairs is underestimated. Regardless, our pan-cancer vaccine is not limited by the population coverage, a common complication in previous studies. Despite the adequate coverage of the EHLA pairs, there was little overlap in mutations with positive EHLA-I and EHLA-II pairs across the cancers. Consequently, cancer-specific EGFR vaccines may be more efficient to produce than a pan-cancer vaccine. To investigate the binding properties of select EGFR epitopes, we created models of some EHLA pairs bound to TCRs. The models demonstrated that the epitopes may bind tightly to HLA molecules while being recognized by TCRs. Our results for this EGFR-specific vaccine may be further validated in preclinical trials with murine cancer models.

Although the current design of AutoPepVax only supports the analysis of missense mutations, future iterations of AutoPepVax could analyze non-missense mutations so that various cancers may be studied. We will also implement updates that improve personalized peptide vaccine composition analysis by including transcriptomic data in our models. Further, we will make AutoPepVax available as a GUI that is easy to download and use.

## 7. Materials and Methods

### 7.1. AutoPepVax Data Collection: Developing Functions to Obtain Epitope Characteristics

AutoPepVax automatically collects the characteristics of mutated epitopes for a given missense mutation. For example, it collects the binding affinity to HLA molecules and antigenicity using various existing in silico tools. AutoPepVax uses these characteristics to produce lists of epitopes likely to induce tumor-specific T cells by employing machine-learning-based models and filtration. AutoPepVax integrates web scraping, Python libraries, and the IEDB-API for data collection. AutoPepVax is coded in Python with 744 lines of code. A description of AutoPepVax’s functions and how to run them is freely available at https://github.com/enricobautista/AutoPepVax (accessed on 21 March 2024).

Each mutated epitope analyzed by AutoPepVax is derived from a missense mutation of a wild-type protein sequence. The binding properties for epitopes are determined in the context of an epitope binding to an HLA molecule. The HLA alleles included in this study are listed in Table 4 When AutoPepVax analyzes missense mutations, each data point is assigned a mutant epitope and an HLA allele, from which all other epitope characteristics are derived. A combination of an epitope and an HLA allele is termed an EHLA pair. Those EHLA pairs associated with MHC-I-restricted epitopes are designated EHLA-I pairs, while those linked to MHC-II-restricted epitopes are called EHLA-II pairs.

For the EHLA pairs, we assessed the binding affinity, measured in nanomolar (nM) units, and rank. Rank is defined as the percentile rank of the binding affinity relative to other EHLA binding affinities. The EHLA-I binding affinity and rank were assessed using MHCflurry 2.0 and NetMHCpan-4.1 BA. MHCflurry 2.0 has been validated by Gartner et al. and Wang et al. [7,27,28,29]. We also calculated the ratio of the wild-type EHLA-I pair rank to the mutant EHLA-I pair rank. For EHLA-II pairs, the rank and binding affinity were measured using NetMHCIIpan-4.0 BA [29]. The stability, a measurement of the binding half-life, was calculated by the NetMHCstabpan 1.0 tool for EHLA-I pairs [30]. AutoPepVax collects binding characteristics using the IEDB-API and the MHCflurry 2.0 Python library.

AutoPepVax uses web scraping or the IEDB-API to automatically collect the remaining EHLA pair characteristics first calculated by various in silico tools. These characteristics and the tools used to calculate them are discussed in the following. Immunogenicity for MHC-I-restricted epitopes was calculated with IEDB’s MHC I immunogenicity tool to predict a peptide’s ability to elicit a T-cell-mediated immune response [30,31]. Each epitope’s antigenicity, or ability to trigger an immune response, was determined using VaxiJen [31]. This tool evaluates the immune response potential of a peptide based on its physiochemical properties using a method known as auto-cross-covariance, rather than relying on the sequence similarity [31]. The toxicity of the epitopes was calculated with ToxinPred. This tool accounts for the dipeptide composition and positions of amino acids to predict whether a peptide is toxic or non-toxic [32]. The ability of MHC-II-restricted epitopes to elicit IFNgamma release by helper T cells was evaluated with the tool IFNepitope [33]. Allergenicity was evaluated by AllerTop v2.0, a tool that identifies probable allergens [34]. Our group evaluated the accuracy of this tool to be 65% [3]. Hydropathicity was calculated as the sum of the corresponding Kyte and Doolittle hydropathy scale for amino acids in positions 4 to *n* − *1* in an epitope, where *n* is the length of the epitope [35]. Other physiochemical characteristics of the epitopes were calculated via the ProtParam tool on the Expasy server [36]. The half-life, isoelectric point, instability index, aliphatic index, and GRAVY score were all recorded according to the tool’s calculations. All the tool links for each characteristic are listed in Supplementary Tables S27 and S28.

### 7.2. AutoPepVax Selection and Ranking of EHLA-I Pairs with Machine-Learning-Based Models

Two machine-learning-based models, a random forest (RF) classification and a linear regression model, were developed to assess the EHLA-I pairs and incorporated into AutoPepVax. The RF classification model separates the EHLA-I pairs into two groups. An identifier (ID) of 1 is assigned to positive EHLA-I pairs likely to induce TILs, and an ID of 0 is assigned to negative non-antigen pairs. The linear regression model assigns high scores to EHLA-I pairs that exhibit favorable binding characteristics. This model ranks EHLA-I pairs according to their binding characteristics. The models were trained with a randomly sampled subset of EHLA-I pairs experimentally determined to be positive or negative [7]. Our training dataset included 109 positive EHLA-I pairs and 654 randomly sampled negative EHLA-I pairs. The complete training data are recorded in Supplementary Table S29. The MHCflurry 2.0 percentile rank, NetMHCpan-4.1 BA rank, NetMHCstabpan prediction, MHCflurry 2.0 wild-type rank to mutant rank ratio, IEDB immunogenicity, VaxiJen antigenicity, and hydropathicity were assessed as potential input features for the RF classification and linear regression model. To discern which input features were best for epitope selection, we performed five-fold cross-validations for models with different combinations of these input features. In five-fold cross-validation, the dataset is split into five subsets. For each fold, one subset is used to train a model, and the rest of the dataset is used to test the trained model. Each fold of the five-fold cross-validations used 80% of the experimental dataset to train the model and 20% to test the model.

We performed a five-fold cross-validation of the RF classification models with each combination of input features. The average accuracy scores, positive predictive values (PPV), and true positive rates (TPR) were recorded for each validation. The RF classification model with input features that had the highest average accuracy was used as AutoPepVax’s RF classification model. We tested the most accurate RF classification model on a list of epitopes from peptide vaccines that were included in clinical trials. The list contained 103 epitopes and included TSAs and tumor-associated antigens (TAAs) [2]. We recorded how many epitopes on the list were predicted to have at least one positive EHLA-I pair. For a comparison of different classification models, we also repeated the cross-validation procedure for the support vector machine (SVM), Gaussian Naive Bayes (GNB), and multi-layer perceptron (MLP) classification models.

We also performed a five-fold cross-validation of the linear regression models with each combination of input features. The average areas under the receiver operator curve (AUC of ROC) were recorded for each validation. The AUC of ROC is a performance metric that assesses the linear regression model’s classification ability across a range of thresholds. We were able calculate the AUC of ROC curve for the linear regression model using the sklearn version 1.3 function “cross_val_score(model, x, y, cv = kf, scoring = ‘roc_auc’)”. This function first computes the linear regression model predictions for a given dataset. Then, the function determines the ROC curve by plotting the true positive rate against the false positive rate for various thresholds that have been applied to the model predictions. Finally, the function calculates the AUC of the ROC curve. The linear regression model with input features that had the highest average AUC of ROC was incorporated into AutoPepVax. We repeated the cross-validation procedure for the logistic, RF, and gradient boosting (GB) regression models to compare the different regression models.

The best RF classification and linear regression models were used to classify and score the EHLA-I pairs. The EHLA-I pairs were grouped by ID and sorted in descending order by score. Then, exclusion criteria were applied to filter the list of EHLA-I pairs by toxicity, half-life, instability index, allergenicity, and ID. The exclusion criteria applied to both EHLA-I and EHLA-II pairs are listed in Table 5. It is important to note that different criteria were used to filter EHLA-I and EHLA-II pairs. Lists of filtered and unfiltered EHLA-I pairs and their characteristics were recorded.

### 7.3. AutoPepVax Filtration of EHLA-II Pairs

For each EHLA-II pair, their immunogenicity, antigenicity, half-life, toxicity, INFgamma, allergenicity, isoelectric point, instability index, aliphatic index, GRAVY score, hydropathicity, NetMHC binding, and NetMHC rank were collected with AutoPepVax. The pairs were then filtered by their immunogenicity, IFNgamma output, antigenicity, toxicity, half-life, instability index, and allergenicity using the exclusion criteria shown in Table 1. Lists of filtered and unfiltered EHLA-II pairs and their characteristics were recorded and are shown in Supplementary Tables S8–S11 and S16–S19.

### 7.4. Applying AutoPepVax to Design of EGFR Peptide Vaccine

**Figure 7:**
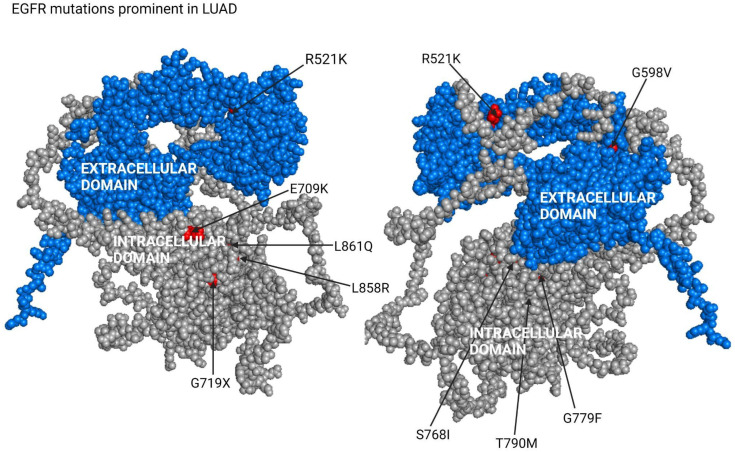
In this figure, arrows indicate the locations of common EGFR missense mutations that occur in LUADs. These missense mutations were assessed using AutoPepVax.

We obtained the 1210-amino-acid-long canonical protein sequence of EGFR from UniProt [37]. Prevalent EGFR missense mutations for glioblastoma multiforme, colorectal adenocarcinoma, and lung adenocarcinoma were obtained from the COSMIC database [38]. EGFR missense mutations in HNSCC were obtained from Nair et al. [19]. The locations of the missense mutations on EGFR for LUAD are shown in Figure 7 We created similar figures for CRAD, GBM, and HNSCC, as shown in Supplementary Figures S4–S6. We input the collected missense mutations and EGFR protein sequence into AutoPepVax. AutoPepVax deposited the files into a specified folder with the filtered and unfiltered lists of EHLA pairs.

### 7.5. Determining Population Coverage of Composite Vaccines

After collecting all EHLA pair data, we utilized our PCOptim-CD program to identify the exclusion criteria restricting the HLA allele coverage [3]. The analysis with PCOptim-CD allowed us to determine how AutoPepVax’s process of epitope selection interacts with the clinical variables to affect the population coverage. We filtered the complete EHLA-I pairs stepwise by toxicity, half-life, instability index, allergenicity, and ID. Similarly, we filtered the complete EHLA-II pairs stepwise by toxicity, half-life, instability index, allergenicity, INFgamma, immunogenicity, and antigenicity. PCOptim-CD produced optimized datasets from each filtered dataset composed of the fewest EHLA pairs required to obtain the maximal population coverage.

### 7.6. Modeling of Peptide–MHC Complexes and TCR Interactions

To confirm the binding affinity of the in silico tools that we used, we created 3D models of select EHLA complexes. For two EHLA-I pairs and one EHLA-II pair, we downloaded their respective HLA molecules from the RCSB Protein Data Bank [39]. We input the HLA molecule and epitope sequences into HPEPDOCK 2.0 [40] to generate each model. Then, HPEPDOCK 2.0 output several confirmations of the input peptide and their associated docking energies. We saved the coordinates of the lowest-energy peptide configuration and the HLA molecule in PDB files. We superimposed the original HLA molecule onto the EHLA complex and saved the 3D coordinates as a PDB file. All models were inspected for clashing in PyMOL [41]. We only created models for the two EHLA-I models bound to TCRs as only a limited number of experimentally validated HLA-TCR complexes were available. TCRmodel was used to create and analyze the TCR binding to top EHLA pairs [42]. Once we found suitable TCRs from the RCSB data bank, we input the sequences of the epitope, the HLA molecule, and the TCR into TCRmodel [39,42]. TCRmodel generated five models and ranked them based on confidence. We inspected the models and downloaded the 3D coordinates of the highest-ranking model with no clash.

## Figures and Tables

**Table 1:** This table includes the names and descriptions of the output files that will populate the folder named after the cancer being studied.

File	Description
CD4 Epitopes.csv	A list of all analyzed EHLA-II pairs and their pertinent characteristics.
CD4 Filtered Epitopes.csv	A filtered list of EHLA-II pairs that meet the exclusion criteria.
CD8 Epitopes.csv	A list of all analyzed EHLA-I pairs and their pertinent characteristics, including ID and score.
CD8 Filtered Epitopes.csv	A filtered list of EHLA-I pairs that meet the exclusion criteria.
Sequence.txt	A list of epitopes for internal use.

**Table 2:** Table of mutations with positive EHLA-I and EHLA-II pairs after filtration for each cancer. Overlapping epitopes imply that some patients harboring these mutations may have a CD4+ and CD8+ T-cell response induced by vaccination.

Cancer	Number of Mutations with Positive EHLA-I and EHLA-II Pairs	Total Mutations	Mutations with Overlapping Epitopes
Glioblastoma Multiforme	1	8	G598V
Colorectal Adenocarcinoma	12	62	R958H, G857R, L707S, E711V, P753L, S442R, G131R, L140V, E709K, R451C, S768G, T710A
Lung Adenocarcinoma	5	11	L861Q, E709K, L858R, G598V, S768I
Head and Neck Squamous Cell Carcinoma	1	15	E967A

**Table 3:** Population coverage results of all epitopes obtained from IEDB.org. (^a^) Projected world population coverage percentage. (^b^) Average epitope/HLA allele hit indicates the average number of epitopes recognized by the population. (^c^) The PC90 is the minimum number of epitopes that are recognized by at least 90% of the population.

Class	EHLA-I Optimized	EHLA-I Filtered	EHLA-II Optimized	EHLA-II Filtered
World Coverage ^a^	98.55%	98.55%	81.81%	81.81%
Average Epitope Hit ^b^	2.3	30.88	1.11	38.74
PC90 ^c^	1.51	11.2	0.55	19.24

**Table 4:** List of MHC Class I and II alleles classified by IEDB. AutoPepVax calculates the binding characteristics of each epitope with all HLA molecules corresponding to the alleles listed in the table.

MHC Class I Alleles	HLA-A*01:01, HLA-A*02:01, HLA-A*02:03, HLA-A*02:06, HLA-A*03:01, HLA-A*11:01, HLA-A*23:01, HLA-A*24:02, HLA-A*26:01, HLA-A*30:01, HLA-A*30:02, HLA-A*31:01, HLA-A*32:01, HLA-A*33:01, HLA-A*68:01, HLA-A*68:02, HLA-B*07:02, HLA-B*08:01, HLA-B*15:01, HLA-B*35:01, HLA-B*40:01, HLA-B*44:02, HLA-B*44:03, HLA-B*51:01, HLA-B*53:01, HLA-B*57:01, HLA-B*58:01
MHC Class II Alleles	HLA-DRB1*01:01, HLA-DRB1*03:01, HLA-DRB1*04:01, HLA-DRB1*04:05, HLA-DRB1*07:01, HLA-DRB1*08:02, HLA-DRB1*09:01, HLA-DRB1*11:01, HLA-DRB1*12:01, HLA-DRB1*13:02, HLA-DRB1*15:01, HLA-DRB3*01:01, HLA-DRB3*02:02, HLA-DRB4*01:01, HLA-DRB5*01:01, HLA-DPA1*01:03/DPB1*02:01, HLA-DPA1*01:03/DPB1*04:01, HLA-DPA1*02:01/DPB1*01:01, HLA-DPA1*02:01/DPB1*05:01, HLA-DPA1*02:01/DPB1*14:01, HLA-DPA1*03:01/DPB1*04:02, HLA-DQA1*01:01/DQB1*05:01, HLA-DQA1*01:02/DQB1*06:02, HLA-DQA1*03:01/DQB1*03:02, HLA-DQA1*04:01/DQB1*04:02, HLA-DQA1*05:01/DQB1*02:01, HLA-DQA1*05:01/DQB1*03:01

**Table 5:** This table lists the exclusion criteria for the filtering of EHLA-I and EHLA-II pairs. After collecting EHLA pairs’ characteristics, AutoPepVax creates a filtered list of EHLA pairs that are not rejected by the exclusion criteria.

Parameter	Exclusion Criteria
Toxicity (EHLA-I and EHLA-II pairs)	Toxin
Half-life (EHLA-I and EHLA-II pairs)	>1 h
Instability Index (EHLA-I and EHLA-II pairs)	>40
Allergenicity (EHLA-I and EHLA-II pairs)	Probable Allergen
IFNgamma (EHLA-II pairs)	Negative
Immunogenicity (EHLA-II pairs)	<50
Antigenicity (EHLA-II pairs)	<0.4
ID (EHLA-I pairs)	=0

## Data Availability

Data are contained within the article and Supplementary Materials.

## Supplementary Materials

The following supporting information can be downloaded at: https://www.mdpi.com/article/10.3390/ph17040419/s1, Supplementary Figures S1–S3: Superimposed images of our EHLA complexes with HLA molecules from the RCSB Protein Data Bank. Supplementary Figure S4: EGFR modeling of mutations prominent in HNSCC. Created with https://biorender.com (accessed on 6 August 2023). Supplementary Figure S5: EGFR modeling of mutations prominent in GBM. Created with https://biorender.com (accessed on 6 August 2023). Supplementary Figure S6: EGFR modeling of mutations prominent in CRAD. Created with https://biorender.com (accessed on 6 August 2023). Supplementary Table S1. This table details the results of 5-fold validation for the classification models. Supplementary Table S2. This table details the results of 5-fold validation for the regression models. Supplementary Table S3. This table includes the mutations studied for each cancer and the total number of MHC-I-restricted epitope–HLA allele pairs before and after filtering. Supplementary Tables S4–S19. These tables include the unabridged filtered and unfiltered lists of epitopes and their characteristics separated by cancer. Supplementary Tables S20 and S21. These tables include the filtered and optimized regional population coverage calculations for MHC-I-restricted epitope–HLA allele pairs. Supplementary Tables S22 and S23. These tables include the filtered and optimized regional population coverage calculations for MHC-II-restricted epitope–HLA allele pairs. Supplementary Tables S24 and S25. These tables include the optimized epitope lists used to find the population coverages. Supplementary Table S26. This table includes the MHC Class II epitopes that were not recognized through IEDB population coverage. Supplementary Tables S27 and S28. These tables include the tool links for each of the MHC Class I and Class II characteristics. Supplementary Table S29. This table includes the experimental epitopes used to train the classifications and regression models. Supplementary PDB Files: The 3D coordinate PDB files for Figure 4, Figure 5 and Figure 6 and Supplementary Figures S1–S3.
