# Supplementary material for: AutoPepVax, a Novel Machine-Learning-Based Program for Vaccine Design: Application to a Pan-Cancer Vaccine Targeting EGFR Missense Mutations
Source: Pharmaceuticals (Basel). 2024 Mar 26;17(4):419. doi: 10.3390/ph17040419 (PMC11053815; doi:10.3390/ph17040419)
Supplement: Supplementary file 1 [file pharmaceuticals-17-00419-s001.zip › Supplementary Figures.pdf]

Supplementary Figures S1-S3 Superimposed images of our EHLA complexes with HLA molecules from the RCSB Protein Data Bank.

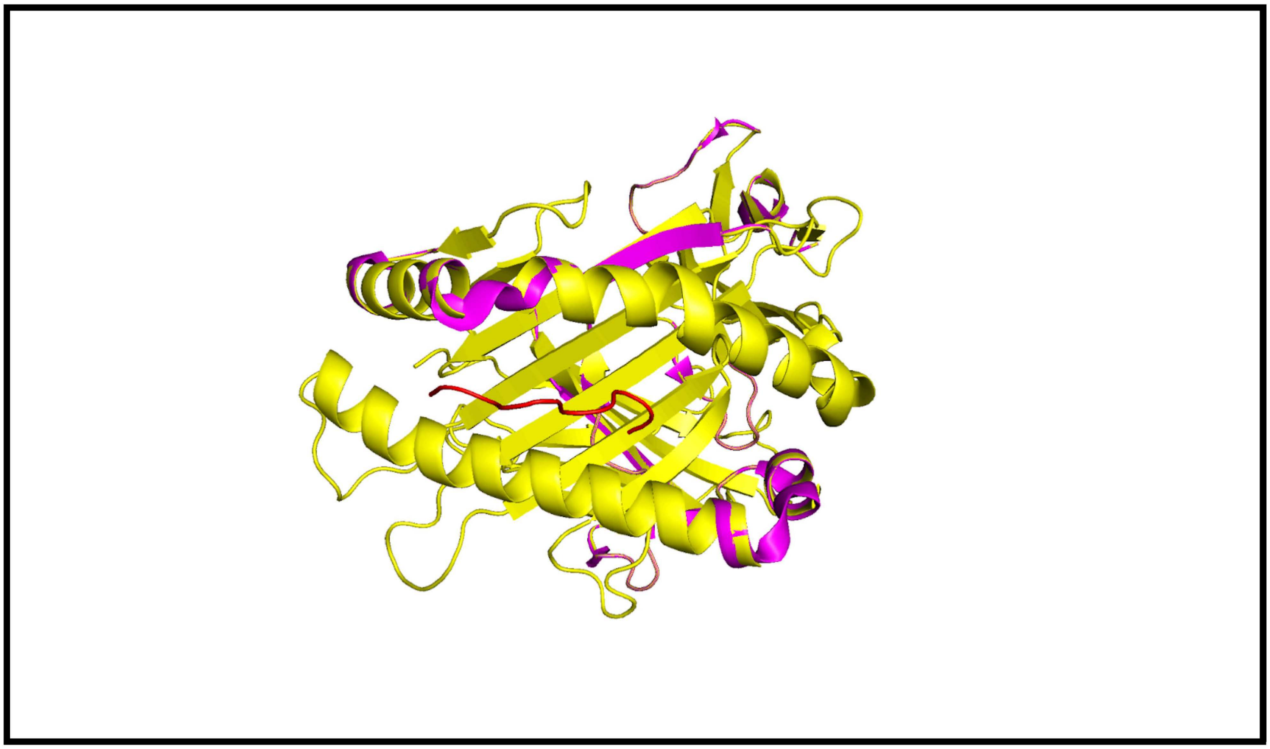

Supplementary Figure S1. VVMGENNTLV glioblastoma multiforme epitope binding to MHC Class I molecule HLA-A*02:06 superimposed with PDB ID: 3OXR (A).

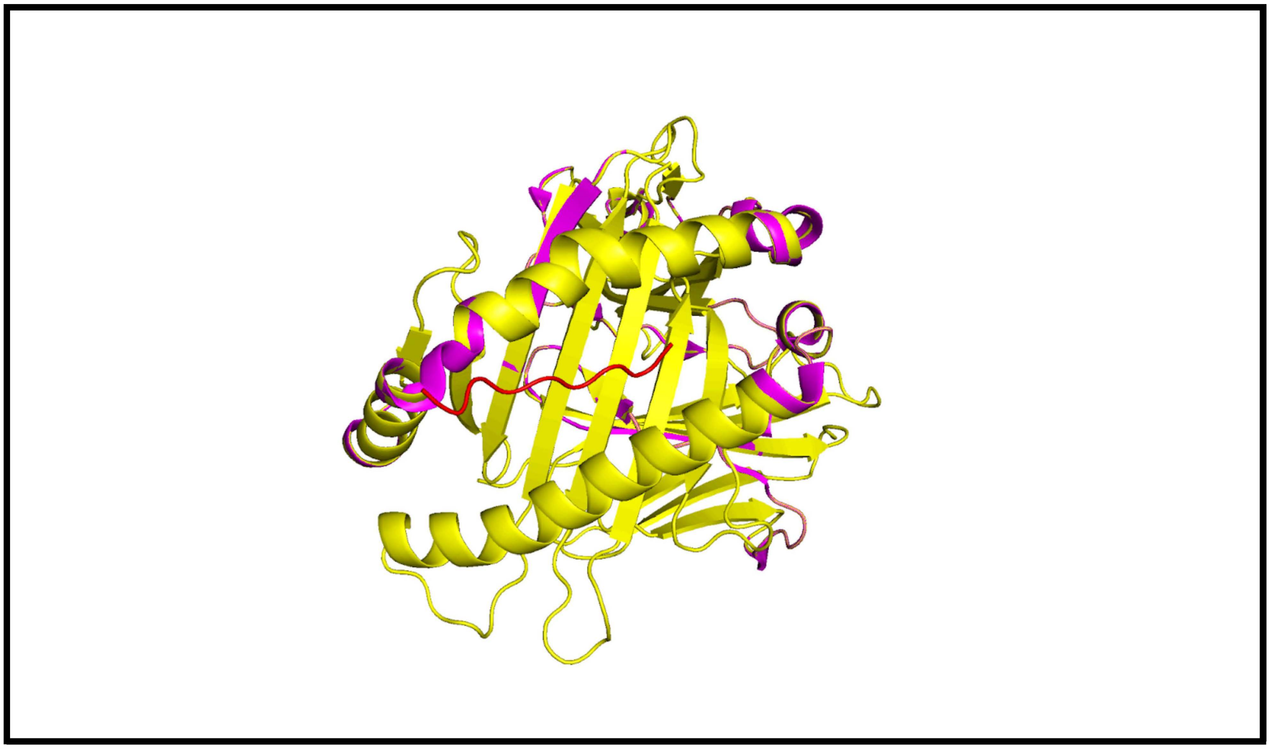

Supplementary Figure S2. ILKETELKK head and neck squamous cell carcinoma epitope binding to MHC Class I molecule HLA-A*03:01 superimposed with PDB ID: 7L1C, A chain.

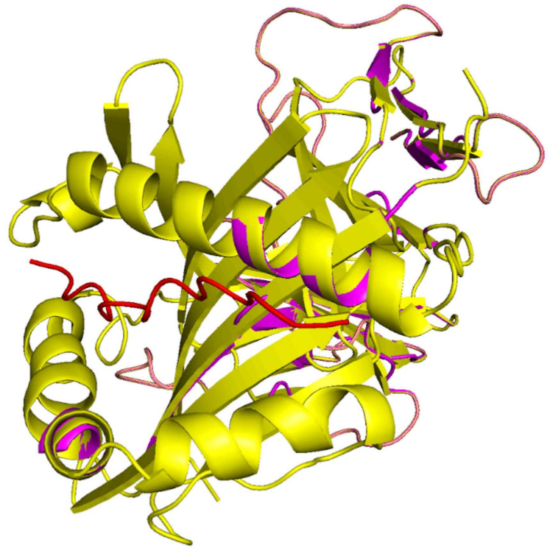

Supplementary Figure S3. ILKKTEFKKIKVLGS colorectal adenocarcinoma epitope binding to MHC Class II molecule HLA-DRB1*04:01 superimposed to PDB: 5JLZ.

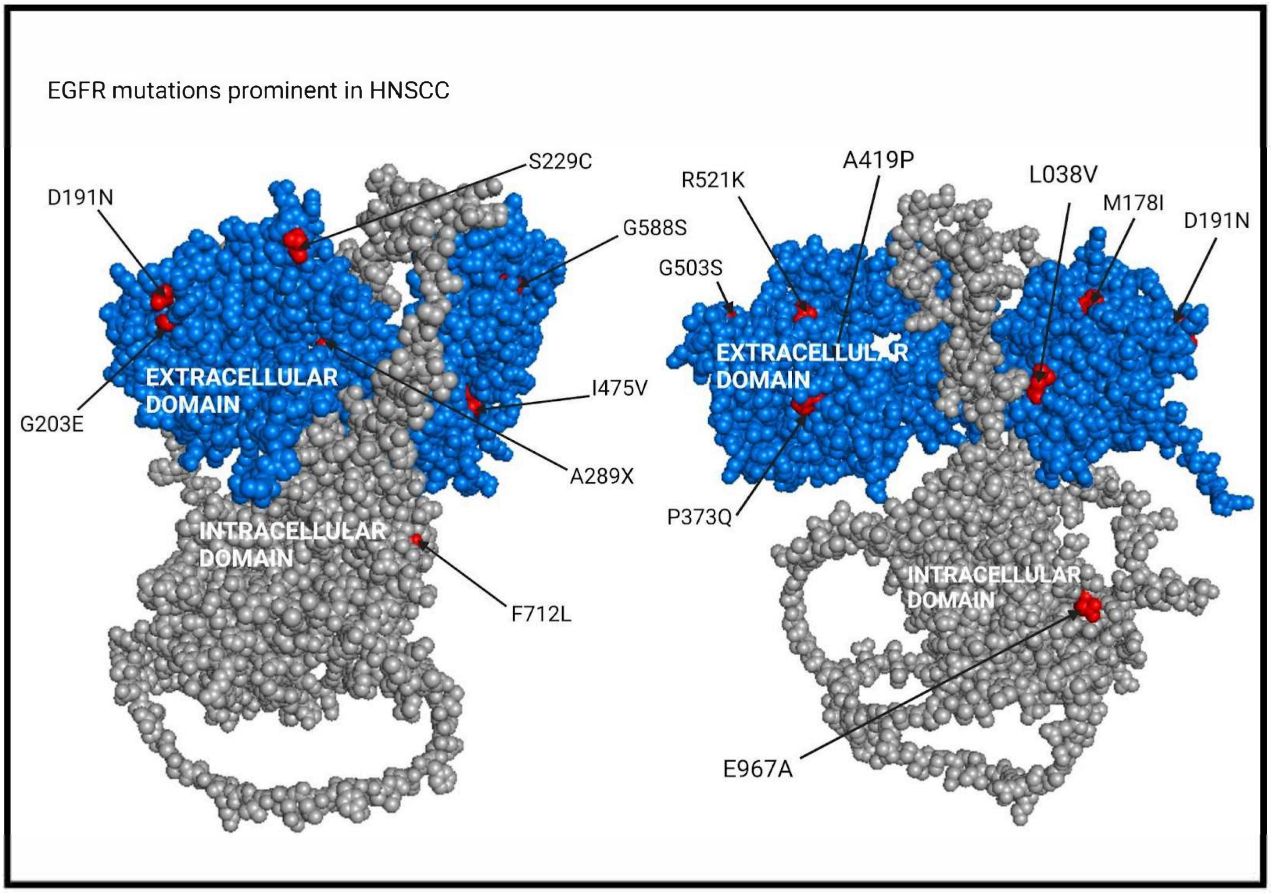

Supplementary Figure S4. EGFR modeling of mutations prominent in HNSCC. Created with https://biorender.com.

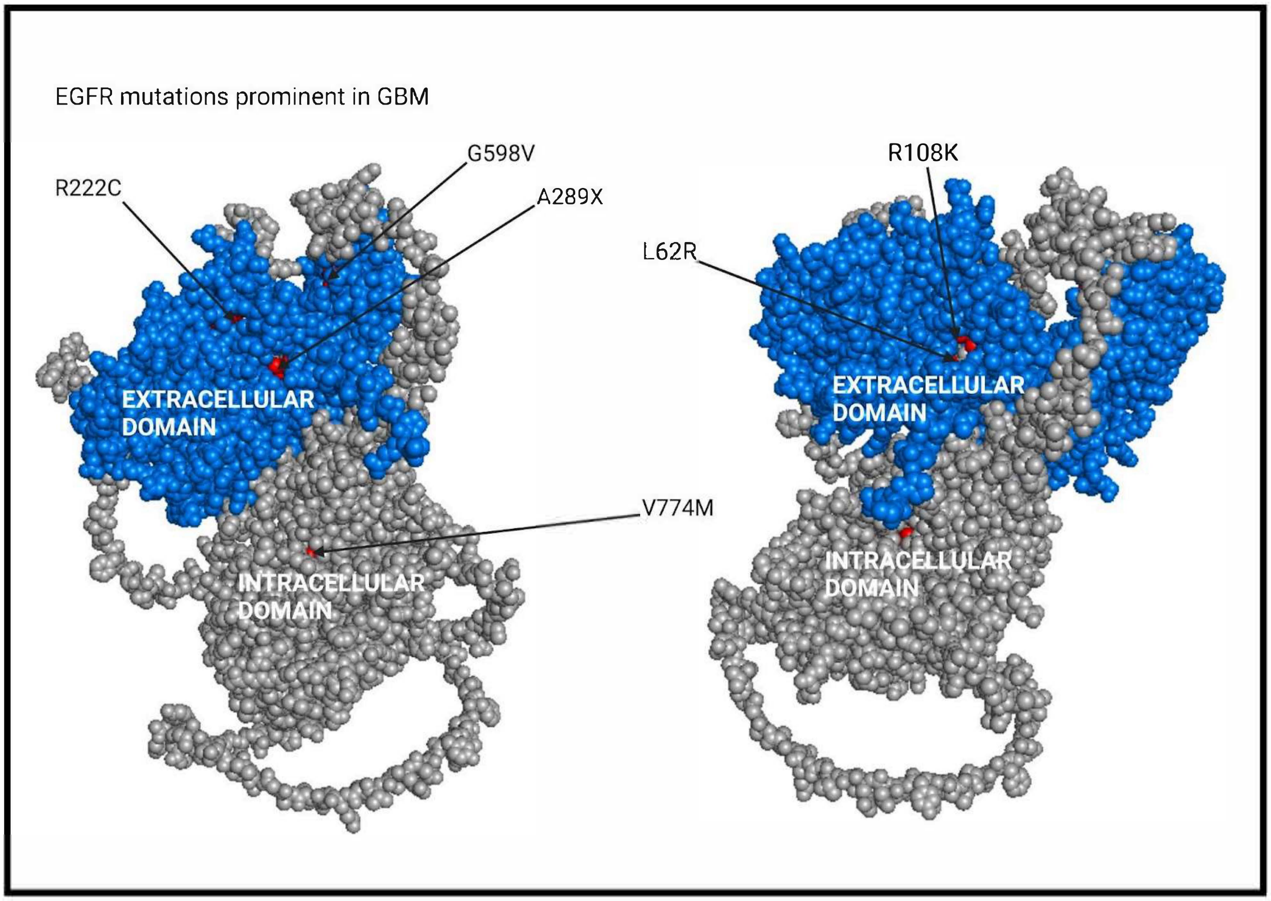

Supplementary Figure S5. EGFR modeling of mutations prominent in GBM. Created with https://biorender.com.

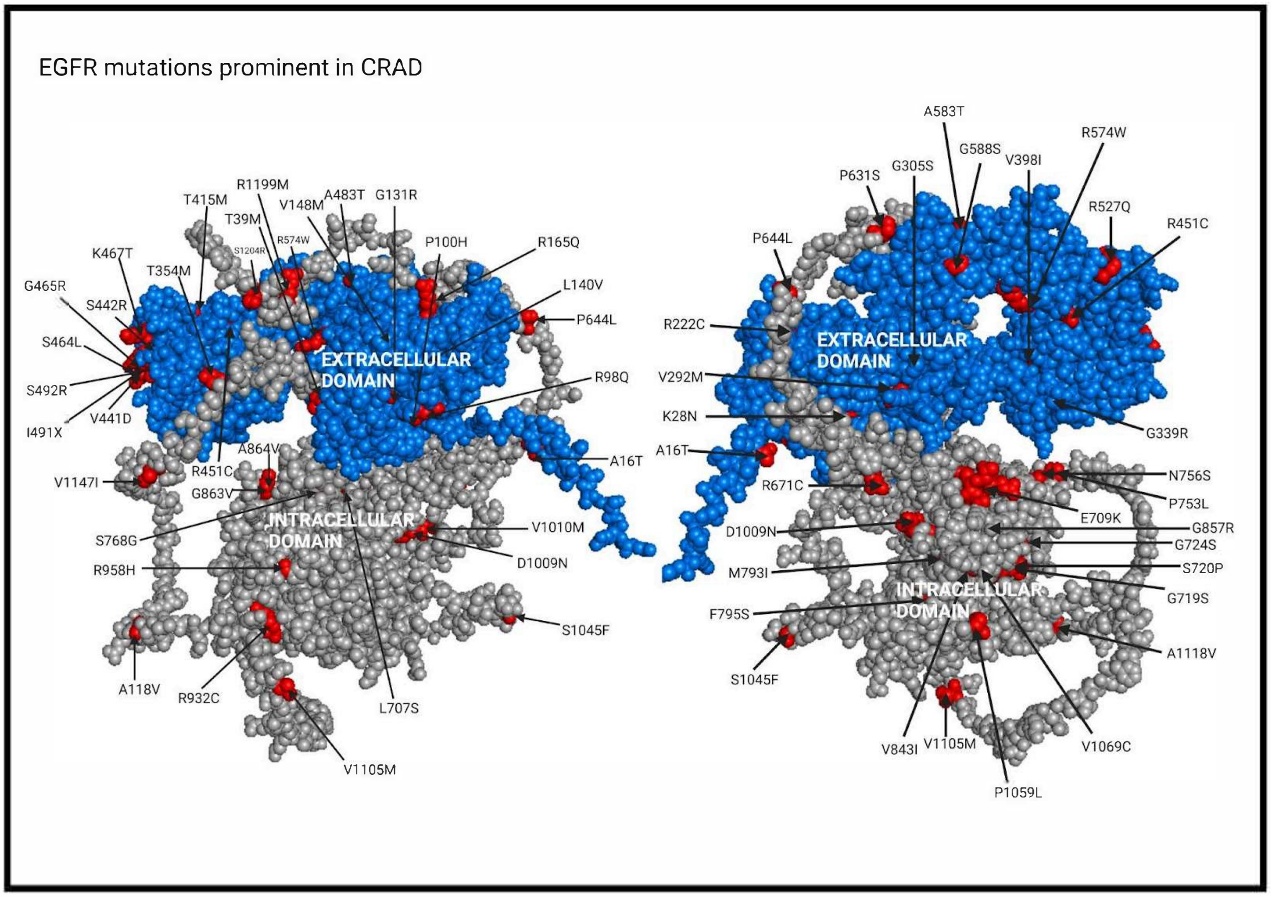

Supplementary Figure S6. EGFR modeling of mutations prominent in CRAD. Created with https://biorender.com.
